# You are more than what you eat: potentially adaptive enrichment of microbiome functions across bat dietary niches

**DOI:** 10.1186/s42523-021-00139-8

**Published:** 2021-12-14

**Authors:** Melissa R. Ingala, Nancy B. Simmons, Miranda Dunbar, Claudia Wultsch, Konstantinos Krampis, Susan L. Perkins

**Affiliations:** 1 Department of Vertebrate Zoology, National Museum of Natural History, Washington, DC USA; 2 Department of Mammalogy, The American Museum of Natural History, New York, NY USA; 3 Division of Invertebrate Zoology, The American Museum of Natural History, New York, NY USA; 4 Department of Biological Sciences, Southern Connecticut State University, New Haven, CT USA; 5 Sackler Institute for Comparative Genomics, The American Museum of Natural History, New York, NY USA; 6 Bioinformatics and Computational Genomics Laboratory, Hunter College, City University of New York, New York, NY USA; 7 Department of Biological Sciences, Hunter College, City University of New York, New York, NY USA; 8 Institute of Computational Biomedicine, Weill Cornell Medical College, New York, NY USA

**Keywords:** Bats, Microbiome, Functional prediction, Dietary ecology, Evolution, 16S rRNA gene

## Abstract

**Background:**

Animals evolved in a microbial world, and their gut microbial symbionts have played a role in their ecological diversification. While many recent studies report patterns of phylosymbiosis between hosts and their gut bacteria, fewer studies examine the potentially adaptive functional contributions of these microbes to the dietary habits of their hosts. In this study, we examined predicted metabolic pathways in the gut bacteria of more than 500 individual bats belonging to 60 species and compare the enrichment of these functions across hosts with distinct dietary ecologies.

**Results:**

We found that predicted microbiome functions were differentially enriched across hosts with different diets. Using a machine-learning approach, we also found that inferred microbiome functions could be used to predict specialized host diets with reasonable accuracy. We detected a relationship between both host phylogeny and diet with respect to microbiome functional repertoires. Because many predicted functions could potentially fill nutritional gaps for bats with specialized diets, we considered pathways discriminating dietary niches as traits of the host and fit them to comparative phylogenetic models of evolution. Our results suggest that some, but not all, predicted microbiome functions may evolve toward adaptive optima and thus be visible to the forces of natural selection operating on hosts over evolutionary time.

**Conclusions:**

Our results suggest that bats with specialized diets may partially rely on their gut microbes to fulfill or augment critical nutritional pathways, including essential amino acid synthesis, fatty acid biosynthesis, and the generation of cofactors and vitamins essential for proper nutrition. Our work adds to a growing body of literature suggesting that animal microbiomes are structured by a combination of ecological and evolutionary processes and sets the stage for future metagenomic and metabolic characterization of the bat microbiome to explore links between bacterial metabolism and host nutrition.

**Supplementary Information:**

The online version contains supplementary material available at 10.1186/s42523-021-00139-8.

## Background

Host-microbe interactions have shaped the ecological and evolutionary history of life on Earth, and there is growing evidence that many animals have adapted to their diets through a combination of physiological adaptations and metabolic pathways encoded by the gut microbiome [1–3]. As a result, many vertebrate groups show gut microbiomes whose taxonomic compositions closely mirror host evolutionary history and dietary strategies [4–6]. Because host diet and evolutionary history are themselves often correlated (i.e., closely related species may share similar diets), it can be challenging to parse the relationship between host diet and evolutionary history in influencing microbiome composition, leaving little consensus on which force is the primary driver in patterning the gut microbiome and whether the strength of these forces varies among host clades [7, 8]. In addition, it can be difficult to extrapolate potentially adaptive functions of animal microbiomes by testing for phylosymbiosis, or the recapitulation of host phylogeny in bacterial community similarity, alone. The majority of studies testing for phylosymbiosis consider only bacterial taxonomy and do not explicitly test any functional hypotheses [but see 6–8]. Because bacterial communities are characterized by a high rate of functional redundancy, phylogenetically unrelated microbial lineages can fulfill similar ecological and metabolic roles [9, 10]. Therefore, different assemblages of bacterial phylotypes within hosts can be functionally convergent even in the absence of taxonomic congruence [11, 12]. For example, three distantly related species of insect-feeding bats from Africa were found to have host-specific assemblages of bacteria, but the predicted functional profiles of the three species' gut communities were largely convergent [13], supporting the observation that similar gut microbiome functions can be fulfilled by different sets of bacteria.

To better understand how microbes have influenced the evolution of their vertebrate hosts, it is essential to understand the functions they provide, as these functions may ultimately become targets of selection. If we consider microbes as aggregates of genes and traits, we might expect ecological filtering to operate more strongly at the level of microbial functions than species identity. We might also expect that nutritionally relevant functions should differ among hosts of different dietary habits, as transitions to new food resources would favor the retention of microbes capable of metabolizing novel food items. It is known that even subtle changes in diet within an animal’s lifetime (e.g., as a result of habitat conversion) are associated with decreased functional capacity in the gut microbiome in primates [14]. Therefore, over evolutionary time, functional repertoires may diverge among hosts with different diets, although this may not unilaterally be the case in host clades that have more depauperate gut communities [12].

To test the hypothesis that microbiome functions should vary among mammals with different diets, we focused on bats as a model system. Bats, the second-most speciose order of mammals, are an ideal system in which to examine functional enrichment among hosts with different diets [15]. Unlike other well-studied host-microbe systems (e.g., primates [16–18] and rodents [19, 20]), the order Chiroptera contains independent dietary radiations into every known terrestrial feeding niche, but especially frugivory, nectarivory, and carnivory [21]. Within this phylogenetic context, it is therefore possible to analyze the enrichment of functional pathways in groups of species that have undergone independent transitions to similar diets. For example, transitions to frugivory occurred in two bat families, the Phyllostomidae and Pteropodidae, independent radiations that happened over millions of years of geographic isolation [22, 23]. Because both of these clades independently switched to a frugivorous lifestyle, it is possible to isolate the influence of host diet away from that of shared evolutionary history in structuring microbiome functions.

To test for enrichment of predicted functional microbial pathways among hosts with different feeding niches, we examined the gut microbiomes of 60 species spanning the full dietary diversity of bats, including insectivorous, frugivorous, omnivorous, sanguivorous (i.e., blood-feeding) and carnivorous species. Using 16S rRNA gene profiling and phylogenetically-informed prediction of bacterial metabolic pathways, we functionally categorized more than 500 individual bat microbiomes and tested for differential enrichment of bacterial metabolic pathways across the five feeding niches. We used both multiple regression of matrices (MRM) and Random Forest decision trees to test the power of microbiome functions to predict host diet and host taxonomic identity.

Finally, we assessed how the enrichment of these inferred pathways is patterned across the bat phylogeny to understand whether they might be visible to natural selection, and thus inform or respond to the evolution of bat hosts. Considering inferred functional pathways as “traits” of the host, we tested this hypothesis by fitting our data to comparative models of trait evolution. Using a host phylogeny, trait values at the tips of the tree are used to compute values at ancestral nodes. Next, expected trait values are simulated under observed models of evolution. Finally, observed trait data can be compared with the trait values expected under each model, and model fit can be assessed using Akaike’s Information Criterion (AIC) [24]. In particular, we were interested in determining if microbiome functional pathways evolve neutrally or in patterns that might invoke natural selection. To test this, we fit our observed functional pathways to four evolutionary trait models: White Noise, Brownian Motion, Early Burst, and Ornstein–Uhlenbeck. These models describe different evolutionary hypotheses about how continuous traits evolve along a phylogeny. The White Noise model assumes traits evolve as random draws from a common distribution, while Brownian Motion (BM) is a neutral model often described as reflecting random drift as a result of mutation as hosts diverge through time. Early Burst (EB) models describe a trait that diversifies rapidly early on in host evolution and slows over time as niches are filled. Finally, the Ornstein–Uhlenbeck (OU) model, like Brownian Motion, is a random walk with an additional parameter that “pulls” traits toward some adaptive optimum, and thus, invokes selection [25].

## Results

**Fig. 1:**
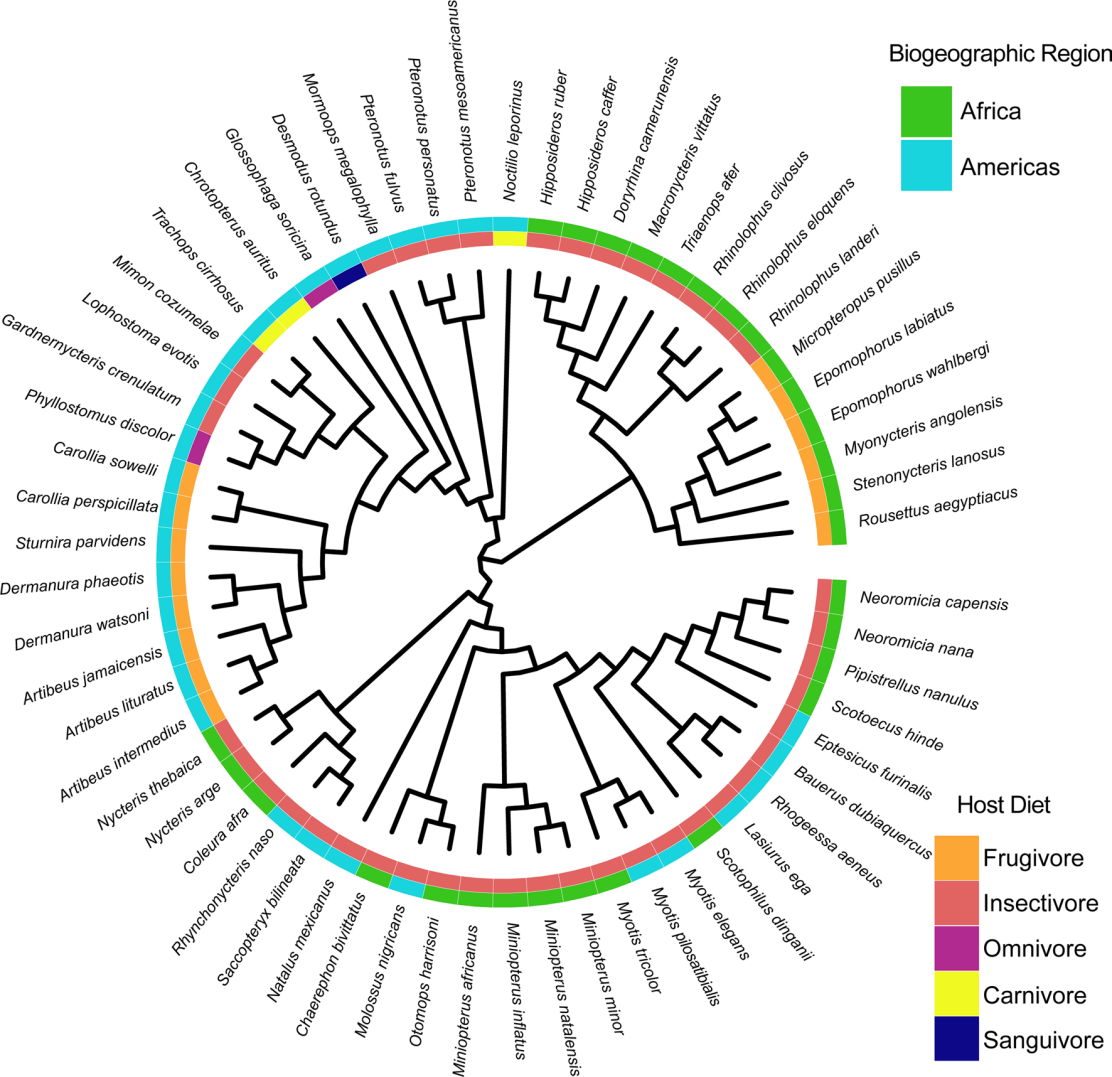
Phylogenetic relationships between hosts sampled in this study. Pruned phylogeny was recovered from VertLife.org (Upham et al. 2019). Biogeographic origin of hosts is indicated in the outermost ring of tiles, while host feeding niche is indicated by the innermost ring of tiles

**Fig. 2:**
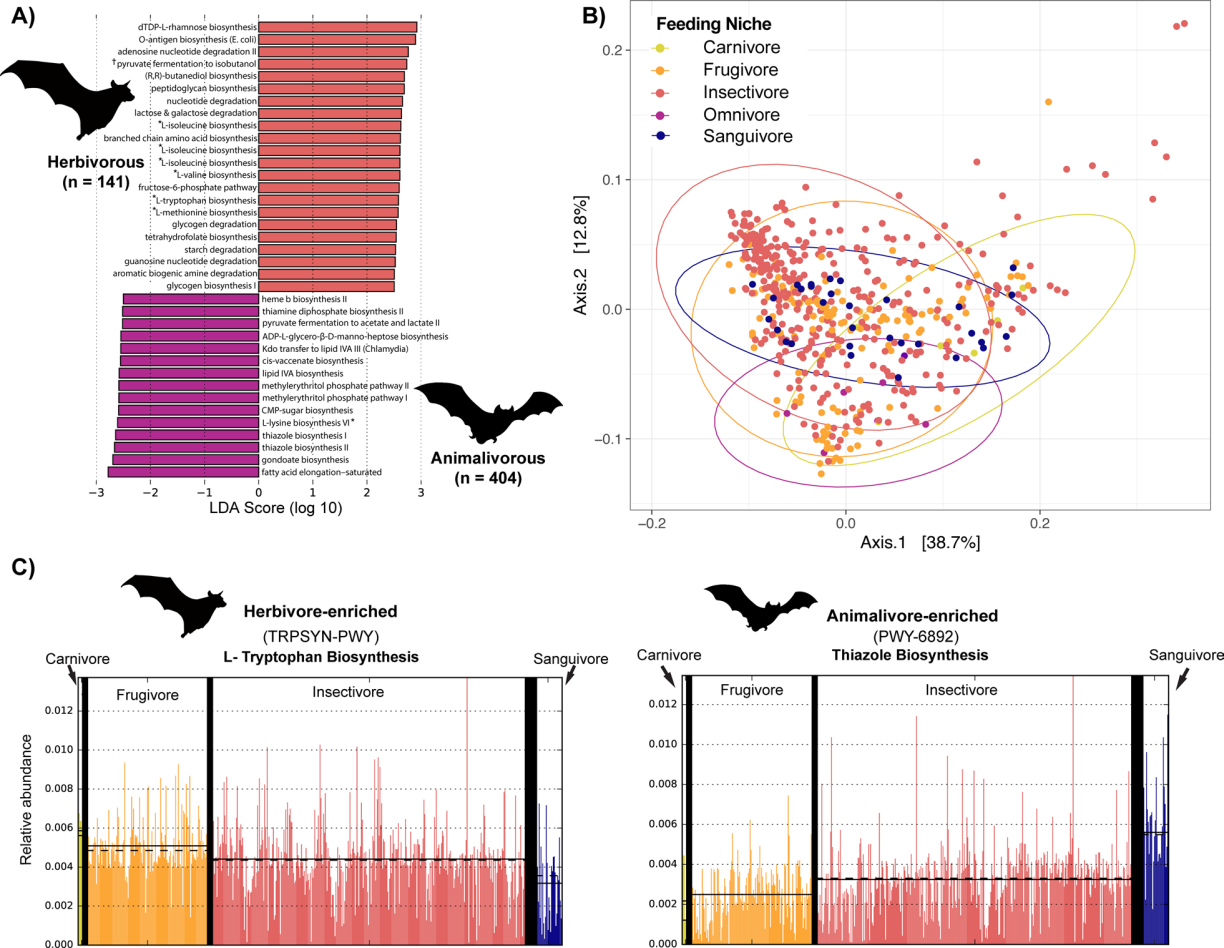
Microbiome functions are differentially enriched across herbivorous and animalivorous bats. **A** Results of LDA-LEfSe analysis of metagenome functions between primarily herbivorous and animalivorous bats (cutoff LDA score ≥ 2.5). The symbol † indicates an engineered pathway, while * indicates a pathway associated with synthesis of an essential amino acid. **B** Principal coordinates analysis of bat metagenome functions, where each dot represents an individual animal’s metagenome. **C** Relative abundance of two functions determined to be differentially enriched in bats of different feeding guilds, where each bar represents one sample. Horizontal lines indicate mean relative abundance within groups. Omnivores are not depicted due to small sample size

The full dataset contained 545 microbiome samples from representatives of 13 families of bats (42 genera, 60 species). This dataset samples all known bat feeding niches and includes instances of repeated independent dietary transitions to frugivory/nectarivory across the order (Fig. 1). Functional prediction with PICRUSt2 resulted in a feature table of 448 MetaCyc pathways [63]. Using PERMANOVA, we found that overall, predicted functional consortia were significantly differentiated by host taxonomy and diet, and that this was true regardless of whether we classified diet using a coarse or fine classification scheme (Fig. 2A, B, Additional file 1: Fig. S1; Table 1). Host taxonomy explained a greater percentage of the variation than diet, although both were significant factors (Table 1). For the pairwise tests, we found that predicted microbiome functions of frugivorous bats were significantly different from those of insectivores, carnivores, and sanguivores, but not different from omnivores (Table 2). Carnivorous bats were highly distinguishable from all other feeding guilds. Omnivorous bats overlapped with frugivorous and sanguivorous bats (Fig. 2B; Table 2) but were distinguishable from strict insectivores and carnivores. The ecologically hyper-specialized vampire bats (sanguivores) had distinctive predicted functional repertoires compared to all other feeding guilds except omnivores (Table 2).

**Table 1:** Result of PERMANOVA of fine and coarse feeding niche on predicted microbiome functions

	Df	Sums of Sq	F_model_	R^2^	*P*_*adj*_
**Fine Niche**	5	0.65	8.67	0.06	**0.001
Host species	55	2.49	3.02	0.24	**0.001
Residuals	484	7.26	–	0.70	
Total	544	10.40	–	1.00	
**Coarse Niche**	2	0.28	9.28	0.03	**0.001
Host species	58	2.87	3.29	0.27	**0.001
Residuals	484	7.26	–	0.70	
Total	544	10.40	–	1.00	

** are significant at the *P* < *0.05* level after Benjamini–Hochberg correction

**Table 2:** Pairwise PERMANOVA results for predicted functions among fine-scale feeding niches

	Sums of Sq	F_model_	R^2^	P-value	*P *_*adj*_
Carnivore ↔ Frugivore	0.103	6.913	0.046	0.001	**0.004
↔ Insectivore	0.114	5.779	0.016	0.006	**0.013
↔ Omnivore	0.060	3.791	0.275	0.008	**0.015
↔ Sanguivore	0.079	5.014	0.125	0.003	**0.009
Frugivore ↔ Insectivore	0.234	12.740	0.025	0.001	**0.004
↔ Omnivore	0.035	2.413	0.016	0.041	0.061
↔ Sanguivore	0.246	16.856	0.090	0.001	**0.004
Insectivore↔ Omnivore	0.058	2.961	0.008	0.028	**0.047
↔ Sanguivore	0.249	12.844	0.032	0.001	**0.004
Omnivore ↔ Sanguivore	0.010	1.203	0.194	0.440	0.507

Comparisons indicated with ** are significant at the *P* < *0.05* level after Benjamini–Hochberg correction

**Fig. 3:**
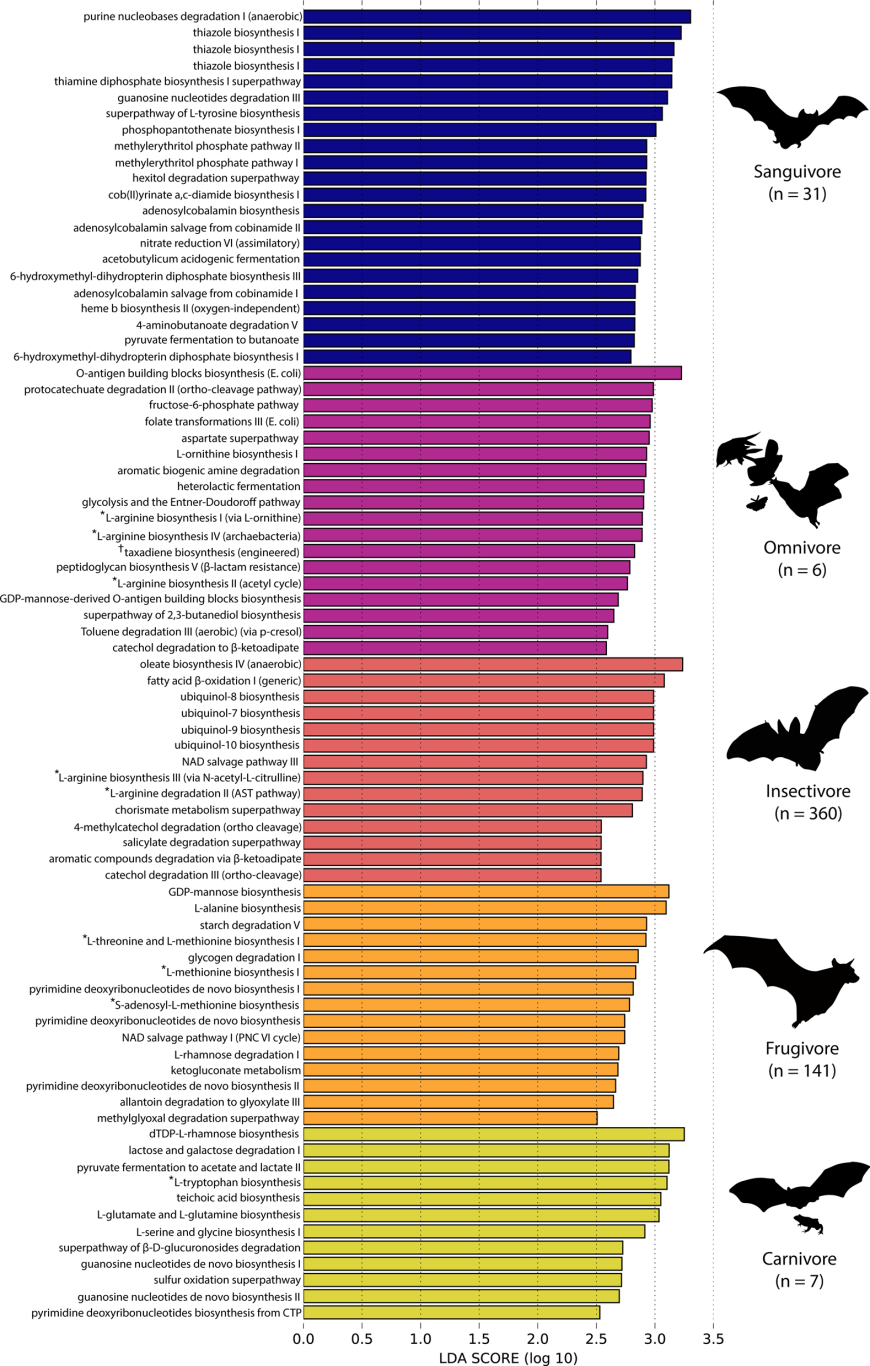
Functional pathways differentially enriched among dietary guilds. LEfSe results for fine scale niche with minimum LDA score cutoff of ≥ 2.5

When we examined which predicted pathways defined each of the dietary niches, LEfSe analysis showed that a total of 37 functional pathways were differentially abundant between primarily animal-feeding and plant-feeding bats (Fig. 2A, Table 3). All of the enriched pathways in animalivorous bats were associated with biosynthesis (93.7%) or generation of precursor metabolites (6.3%), while pathways enriched in herbivorous bats were split between biosynthesis (62.0%), degradation, utilization, and assimilation functions (33.3%), and generation of precursor metabolites (4.7%). Six of the pathways enriched in herbivorous bats were associated with proteinogenic amino acid biosynthesis, specifically the production of the essential amino acids isoleucine, valine, tryptophan, and methionine [64]. Figure 2C (left) depicts the differential abundance of one of these pathways, the L-tryptophan biosynthesis pathway, among feeding niches. Pathways enriched in animal-feeding bats were more general, and were split among fatty acid, amino acid, and secondary metabolite processing pathways (Fig. 2A, Table 3). The relative abundance of one animalivore-enriched pathway, the thiazole biosynthesis pathway, is shown in Fig. 2C (right). Another pathway found to be enriched in frugivores was determined to be PWY-7111, an engineered pathway not known to occur naturally in any bacterial species. The contributing bacterial ASV for this pathway likely did not match closely to a known reference microorganism during the PICRUSt2 predictions (Fig. 2A; Table 3). We also performed LEfSe differential abundance analysis on the fine-scale niche groupings. The results were consistent with the coarse analysis, with some additional pathways contributing to the observed differences among dietary ecologies (e.g., ketogluconate metabolism for frugivores, cobinamide salvage in sanguivores) (Fig. 3).

**Table 3:** Differentially enriched metagenome functions recovered from LEfSe analysis

MetaCyc Pathway	Superpathway	Enrichment	LDA Score	Wilcoxon *P*
DTDPHRAMSYN_PWY	Carbohydrate Biosynthesis	Herbivorous	2.92	1.27E−14
OANTIGEN-PWY	Carbohydrate Biosynthesis	Herbivorous	2.90	8.91E−15
SALVADEHYPOX-PWY	Nucleoside and Nucleotide Degradation	Herbivorous	2.76	2.10E−07
†PWY-7111	Engineered	Herbivorous	2.74	6.98E−09
P125-PWY	Other Biosynthesis	Herbivorous	2.69	7.95E−20
PWY-6471	Cell Structure Biosynthesis	Herbivorous	2.69	8.12E−07
PWY-6353	Nucleoside and Nucleotide Degradation	Herbivorous	2.66	6.18E−07
LACTOSECAT-PWY	Carbohydrate Degradation	Herbivorous	2.64	1.40E−13
*PWY-5101	Amino Acid Biosynthesis	Herbivorous	2.63	1.10E−07
BRANCHED-CHAIN-AA-SYN-PWY	Amino Acid Biosynthesis	Herbivorous	2.62	3.68E−09
*PWY-5103	Amino Acid Biosynthesis	Herbivorous	2.61	2.09E−09
*ILEUSYN-PWY	Amino Acid Biosynthesis	Herbivorous	2.61	8.36E−08
*VALSYN-PWY	Amino Acid Biosynthesis	Herbivorous	2.61	8.36E−08
P124-PWY	Fermentation	Herbivorous	2.59	3.39E−09
*TRPSYN-PWY	Amino Acid Biosynthesis	Herbivorous	2.58	7.93E−07
*HSERMETANA-PWY	Amino Acid Biosynthesis	Herbivorous	2.58	1.23E−06
GLYCOCAT-PWY	Polymeric Compound Degradation	Herbivorous	2.55	7.96E−06
PWY-6612	Cofactor, Prosthetic Group, Electron Carrier, and Vitamin Biosynthesis	Herbivorous	2.54	7.51E−09
PWY-6737	Polymeric Compound Degradation	Herbivorous	2.53	2.33E−05
PWY-6608	Nucleoside and Nucleotide Degradation	Herbivorous	2.52	8.16E−05
PWY-7431	Amine and Polyamine Degradation	Herbivorous	2.50	3.29E−08
GLYCOGENSYNTH-PWY	Carbohydrate Biosynthesis	Herbivorous	2.50	1.83E−05
HEMESYN-PWY	Cofactor, Prosthetic Group, Electron Carrier, and Vitamin Biosynthesis	Animalivorous	2.50	0.0165
PWY-6895	Cofactor, Prosthetic Group, Electron Carrier, and Vitamin Biosynthesis	Animalivorous	2.51	9.74E−09
PWY-5100	Fermentation	Animalivorous	2.52	0.013
PWY0-1241	Carbohydrate Biosynthesis	Animalivorous	2.55	1.68E−06
PWY-6467	Cell Structure Biosynthesis	Animalivorous	2.55	0.00052
PWY-5973	Fatty Acid and Lipid Biosynthesis	Animalivorous	2.55	1.88E−05
NAGLIPASYN-PWY	Cell Structure Biosynthesis	Animalivorous	2.58	6.92E−05
PWY-7560	Secondary Metabolite Biosynthesis	Animalivorous	2.58	0.00044074
NONMEVIPP-PWY	Secondary Metabolite Biosynthesis	Animalivorous	2.58	0.0004
PWY-1269	Carbohydrate Biosynthesis	Animalivorous	2.59	5.64E−05
*PWY-5097	Amino Acid Biosynthesis	Animalivorous	2.61	0.0004
PWY-6892	Cofactor, Prosthetic Group, Electron Carrier, and Vitamin Biosynthesis	Animalivorous	2.64	3.33E−08
PWY-6891	Cofactor, Prosthetic Group, Electron Carrier, and Vitamin Biosynthesis	Animalivorous	2.66	1.09E−09
PWY-7663	Fatty Acid and Lipid Biosynthesis	Animalivorous	2.70	2.92E−09
FASYN-ELONG-PWY	Fatty Acid and Lipid Biosynthesis	Animalivorous	2.78	3.86E−09

All LDA scores were retained only where LDA ≥ 2.5 and are shown rounded to the second decimal place. Wilcoxon test was considered to be significant if *P* ≤ *0.05*. † is an engineered metabolic pathway, while * notes a synthesis pathway for an essential or conditionally essential amino acid

Matrix regressions (MRM) run on the full dataset determined that only the patristic distance (i.e., phylogenetic distance between hosts) was predictive of microbiome functional dissimilarity (MRM *P*_phylo_ = 0.01) while ecological distances computed from EltonTraits quantitative data were not significantly predictive (MRM *P*_ecol_ = 0.38). However, matrix regression analysis requires merging all within-species replicates to create a distance matrix based on averaged values for each species. To account for high amounts of inter-individual variation in microbiomes, we also subjected the data to Random Forest analysis to test the predictive power of metagenome functions on an individual sample, rather than whole-species, basis.

**Fig. 4:**
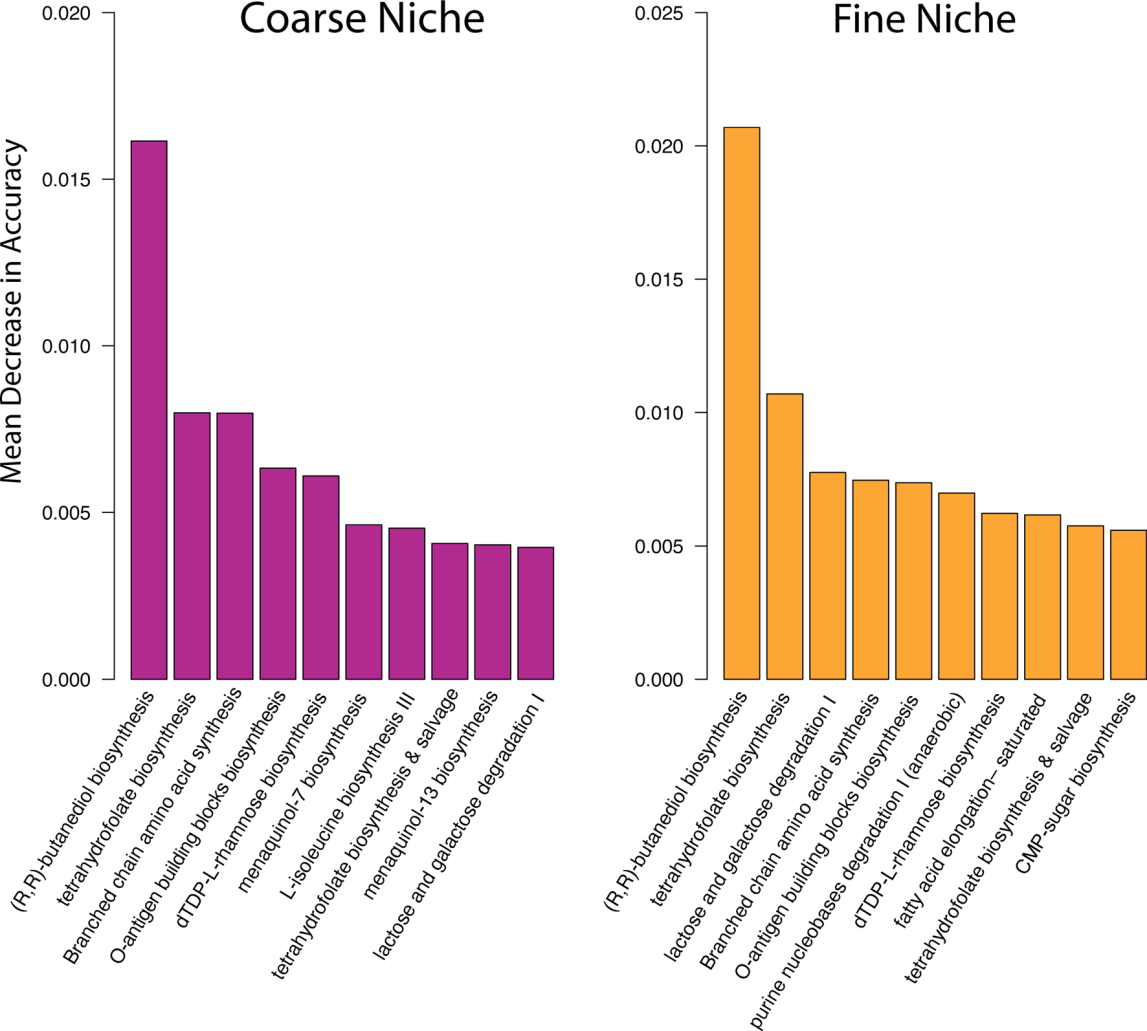
Top ten most important functional pathways for the coarse (purple) and fine scale (orange) classification models. Variable importance was determined by ranking the mean decrease in accuracy for each of the metagenome functions used to create the random forest classifiers. Coarse classification scheme: animalivorous vs. herbivorous, Fine classification scheme: sanguivorous, omnivorous, insectivorous, frugivorous, carnivorous

Random Forest (RF) analyses were conducted to test the ability of metagenomic functions to classify bats into dietary guilds as well as host family and genus. For the coarse (animalivorous vs. herbivorous) niche classification model, the out-of-bag error rate was 13.2%. Within-class error varied according to host niche; the model performed particularly well at identifying primarily animalivorous bats based on metagenome functions, but less so for primarily plant-feeding animals, and very poorly for omnivores (Table 4). The fine niche model performed slightly worse, with an OOB of 15.6%. Similarly, the model performed best at predicting the insectivorous classifications, followed by frugivorous, and struggled substantially to predict omnivores, carnivores, and sanguivores (Table 5). Models for predicting host family and genus performed poorly, with out-of-bag error rates of 49.4% and 58.4%, respectively (Additional file 4: Table S3, Additional file 5: S4). We tested the accuracy of the RF niche models using leave-one-out cross-validation, a resampling procedure used to estimate how a model is expected to perform in general when used to make predictions on data not used during the training of the model. Cross-validation on 500 trees produced an accuracy rate of 86.6% (Kappa = 0.626) for the coarse classification model and 84.2% (Kappa = 0.650) for the fine classification model. We next sorted the functional variables by mean decrease in model accuracy (i.e. variable importance in training the model). The resulting top ten most informative features for classifying host diets are shown in Fig. 4.

**Table 4:** Confusion matrix for the coarse niche random forest model

	Animalivorous	Omnivorous	Herbivorous
Animalivorous	386	0	12
Omnivorous	2	0	4
Herbivorous	54	0	87

Within-class error rates were 3.0% for Animalivores, 100% for omnivores, and 38% for herbivores

**Table 5:** Confusion matrix for fine-scale niche random forest model

	Carnivore	Frugivore	Insectivore	Omnivore	Sanguivore
Carnivore	0	0	6	0	0
Frugivore	0	92	49	0	0
Insectivore	0	14	346	0	0
Omnivore	0	4	2	0	0
Sanguivore	0	1	8	0	22

Within-class error rates were 100% for carnivores, 34.8% for frugivores, 3.9% for insectivores, 100% for omnivores, and 29% for sanguivores

**Fig. 5:**
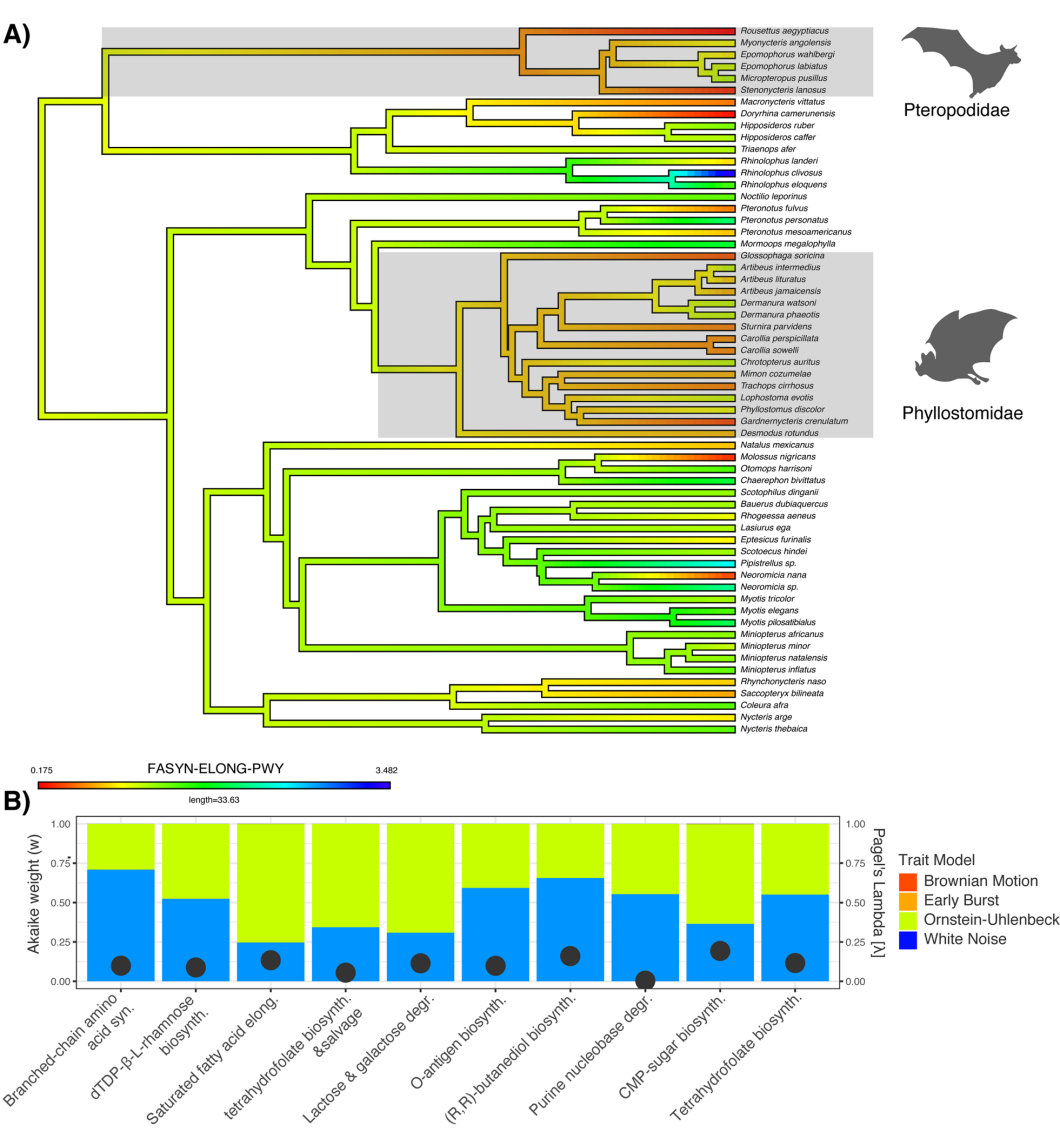
Phylogenetic comparative analyses. **A** Host phylogeny colored by average functional pathway abundance for FASYN-ELONG-PWY. Shaded boxes highlight two clades with independent transitions from insectivory to other dietary guilds (Pteropodidae, Phyllostomidae). **B** Weighted AICs for four evolutionary models and lambda estimates for the ten pathways most informative for discriminating among diet guilds, shown as overlaid grey points

Phylogenetic comparative analyses were performed on the ten most informative functional pathways identified by random forest analysis (Fig. 4). Our sampling of the clade encompassed 13 families, representing about 60% of the family-level diversity of extant bats [65]. The enrichment or depletion of predicted microbiome pathways was not distributed randomly with respect to host phylogeny; for example, the saturated fatty acid elongation pathway was selectively underenriched in the Pteropodidae and Phyllostomidae (Fig. 5A). To test for phylogenetic signal in pathway enrichment, we calculated Pagel’s λ for all ten metagenomic pathways. All pathways had low phylogenetic signal in general, with P164-PWY (purine nucleobase degradation) having λ statistically equivalent to zero. The pathways OANTIGEN-PWY (O-antigen biosynthesis pathway) and BRANCHED-CHAIN-AA-SYN-PWY (branched chain amino acid synthesis superpathway) both had low phylogenetic signal with λ = 0.1. The pathways PWY-6612 (tetrahydrofolate biosynthesis superpathway), LACTOSECAT-PWY (lactose and galactose degradation superpathway), and DTDPRHAMSYN-PWY (dTDP-β-L-rhamnose biosynthesis) all had λ = 0.12. The pathways with the highest phylogenetic signal were the FASYN-ELONG-PWY (saturated fatty acid elongation, λ = 0.13), P125-PWY ((R,R)-butanediol biosynthesis, λ = 0.16), and PWY-1269 (CMP-3-deoxy-D-manno-octulosonate biosynthesis, λ = 0.19) respectively (Fig. 5B).

In terms of model fit, all AIC weight was split between the OU and White Noise models, with Brownian Motion and Early Burst models receiving none of the AIC weight for any pathway. The White Noise model received > 50% of the weight for 5 of the 10 pathways tested, with another 2 models sharing 50–50 split between White Noise and OU models. The OU model received > 50% of the weight for only three pathways– saturated fatty acid elongation, tetrahydrofolate biosynthesis and salvage, and lactose and galactose degradation (Fig. 5B).

## Discussion

Our current understanding of host-microbe interactions in wild mammals is mostly limited to observations of phylosymbiosis between host clades and their microbiotas. While these tests are a necessary and foundational step in symbiosis research, the true impacts of microbial symbionts on host fitness and evolution cannot be fully understood without more explicit inventories of the functions that these communities contribute to their hosts. In this study, we found that bats with different dietary specializations have microbiomes enriched with potentially adaptive predicted functions (Table 3), and that these functions can be used to predict the dietary classification of the host (Tables 4, 5). When we considered bats as either primarily herbivorous or animalivorous, very few predicted functions could significantly discriminate among the groups. However, of the pathways that were found to be enriched in herbivorous (i.e. fruit- or nectar-feeding) bats, several were pathways associated with the production of the essential amino acids methionine, valine, isoleucine, and tryptophan (Fig. 2A; Table 3). Essential amino acids are those than cannot be synthesized de novo by the host; they must either already be present in the diet or produced through microbial metabolism and absorbed through the host intestine [26, 27]. Essential amino acids may be particularly limiting nutrients for obligate frugivores; fruits consumed by fruit bats are deficient in protein compared with insects [28, 29], such that existing on a diet primarily consisting of fruit may pose nutritional challenges that can be partially overcome by the metabolic contributions of symbiotic microbes. In a mouse model, it was shown that gut microbes can provision essential amino acids when hosts are fed on protein-deficient diets [30], lending further evidence that gut bacteria play essential nutritional roles in their hosts.

While the exact fruits consumed by many frugivorous bat species are unknown, phyllostomid bats are known to feed on *Piper* and *Ficus* fruits, which are considered to be nutritionally poor food resources [28]. The bacterial genus *Erwinia* was prevalent in the microbiotas of Belizean fruit bats; members of this genus are known to provide supplemental protein to herbivorous adult olive flies, suggesting that they may fulfill a similar role in the microbiomes of fruit bats from Belize [31]. Fruit consumed by the pteropodid species *Micropteropus pusillus* and *Epomops buettikoferi* were shown to contain 3.3% protein by dry mass, indicating that African fruit bats feed on nutritionally poor fruits as well [29]. In African fruit bats, the bacterial genus *Gemella* was enriched compared to insectivores [11]. This genus is predicted to be capable of synthesizing methionine according to the Kyoto Encyclopedia of Genes (KEGG), and may partially explain the enrichment in L-methionine pathways we observed in frugivores (Fig. 3). Other functions enriched in herbivorous bats were related to carbohydrate degradation (e.g., glycogen and starch), as well as biosynthesis of the B-vitamin folate. Enrichment in these bacterial pathways may be complementary to the nutritional composition of various fruits, which are made up primarily of water and simple carbohydrates with relatively few proteins, vitamins, and minerals [28, 32, 33].

Differential enrichment analysis detected more functional pathways discriminating among dietary groups when classified more finely (Fig. 3). Notably, in our sample of 23 vampire bat (*Desmodus rotundus*) microbiomes, we found many pathways related to cofactor and vitamin biosynthesis, inorganic nutrient metabolism, and amine degradation to be enriched (Fig. 3). This is consistent with previous findings by Zepeda-Mendoza et al. (2018), which showed enrichment of microbial genes related to cofactor and vitamin metabolism, siderophore biosynthesis (important for handling iron and heme), and amino acid metabolism [2]. To link these functions to bacterial phylotypes present in the vampire bat microbiome, we find that vampire bat gut microbiotas are characterized by high relative abundance of Peptostreptococcaceae, which have the rare ability among microbes to ferment amino acids [34, 35]. Blood is 78% liquid, but its solid cellular phase contains 93% proteins and less than 1% carbohydrates [36], suggesting that members of this bacterial clade may assist in protein processing in the vampire bat host.

Overall, animalivorous bats had metagenomes that were characterized by vitamin, proteinogenic amino acid, fatty acid, and carbohydrate synthesis (Table 3). This more generalized suite of microbial functions is likely a byproduct of energetic demands on insectivorous hosts. Insect-eating bats rely on recently consumed exogenous resources to fuel flight, which may possibly select for microbes which can generate other, non-combustible metabolites for later use by the host [37, 38].

In addition to identifying specific pathways associated with the feeding habits of these species, we wanted to know how predictive overall functional composition was of dietary guild. Our random forest models performed well at predicting host diet, with accuracy rates between 80 and 85% regardless of whether we classified diet using a coarse or fine classification scheme. The models were best at predicting insectivorous or primarily animalivorous species based on their gut microbiomes but were substantially worse at predicting frugivores and omnivores. It is important to note that many dietary specializations, including frugivory and nectarivory, are more labile than previously thought [39–41]. For instance, some species of bats are known to occasionally take insects despite being considered “frugivores” [42], so rather than existing as discrete, closed niches, many bat species probably fall along a spectrum running from primarily plant-feeding, to omnivorous, to primarily animal-feeding. In light of this view, it is unsurprising that the random forest models failed to correctly identify omnivores 100% of the time. Omnivorous microbiomes are not likely characterized by their own suites of functions per se. Rather, they are more likely functionally intermediate between strict insectivores and frugivores, which the PCoA of predicted metagenome functions supports (Fig. 2B). The matrix regressions did not detect a meaningful correlation between host metagenomic distances and diet; however, given the high level of within-species microbiome variation in bats [11, 12, 43], it is likely that averaging functions within species so that they match the taxon-level dietary data from EltonTraits introduces inappropriate levels of noise to the distance-based analysis. Taken with our random forest results, we conclude that host diet and microbiome functions may be related on a per-sample rather than per-taxon basis. The phylogenetic matrix regression also recovered a relationship between host phylogeny and microbiome function, suggesting that overall functional profiles may be related to host evolutionary history. However, the MRM method requires collapsing all of the microbiome functional variation into patristic distances that can obscure more fine-scale patterns. Random Forest models were unable to predict host family or genus membership using microbiome functions alone (Additional file 4: Table S3, Additional file 5: Table S4), suggesting that while functions are characteristic of host diet, they are not able to discriminate among related hosts.

By contrast, the comparative phylogenetic analyses, which were performed on individual pathways rather than distances, detected very low phylogenetic signal in all of the tested pathways, with the data for most pathways best fitting a white noise, or phylogeny independent, model of trait evolution (Fig. 5B). However, three critical metabolic superpathways dealing with unsaturated fatty acid elongation, folate biosynthesis, and lactose catabolism were more heavily weighted toward an OU model of evolution (Fig. 5B). The OU model differs from a Brownian Motion model in that a stochastically varying trait is assumed to evolve toward an optimal value rather than neutrally along the phylogeny [44]. While we cannot say for certain whether the pathways fitting the selection-invoking evolutionary model are optimized to host ecology, when these pathways are mapped onto the host phylogeny, it is clear that their enrichment or depletion is mostly clustered in the clades that have experienced independent transitions away from insectivory, the Phyllostomidae and the Pteropodidae (Fig. 5A). Taken together with the results of the differential enrichment analyses, we hypothesize that a subset of metagenome functions may respond to selective pressures imposed by host diet, such that hosts with nutritionally challenging diets favor the retention of microbial functions that help facilitate their metabolic needs. A major caveat of this approach is that microbiome functions need to be heritable to be considered as traits of the host. Current evidence for vertical transmission is lacking for mammals, but there is some evidence that wild animals have a higher proportion of heritable gut bacteria than previously thought [45]. Given that the gastrointestinal traits governing this filter have a genetic, and therefore potentially heritable, basis [46], we also suggest that some microbiome members may be considered as “functionally inherited” if microbial metabolites, rather than species, are the actual targets of host selection [47].

While our data suggests a role for microbes in host dietary evolution, our results come with some important limitations. One consideration is that microbiomes can shift in response to seasonal variation in diet which would not be captured by our cross-sectional sampling scheme. In addition, although PICRUSt2 has been shown to perform well at predicting functional pathways, this algorithm fundamentally relies on the completeness of microbial gene databases that link pathways with bacterial species. Because the bat microbiome is relatively poorly characterized, some taxa may not have particularly close matches in the database. In addition, amplicon-based functional predictions assume that all pathways are active. Direct inventories with shotgun metagenomic methods can be applied in the future to address database completion issues, while metatranscriptomics can address which members of the community are actively transcribing vs. dead or dormant (e.g., [48]). Metabolomic and isotopic techniques can further help to pinpoint specific metabolites contributed by the host’s own physiological process versus those created by microbes [30, 49].

Our results suggest that bats across various feeding niches may rely on their gut symbionts to fulfill essential metabolic roles that are related to host dietary ecology, though the strength of this dependence likely depends on the level of host dietary specialization. These results re-contextualize our understanding of host-microbe interactions within bats. Two recent studies did not detect a signal of phylosymbiosis among bats and their gut microbiotas, perhaps suggesting that it is unlikely that bats depend on their microbiomes as much as other vertebrates because the energetic demands of flight make maintaining these associations too costly [11, 12]. Our results demonstrate that numerous bacterial pathways—which may be encoded by a taxonomically diverse set of organisms—are correlated with dietary specialization in bats, suggesting at least some role for microbes in their ecological diversification. Questions still remain regarding the strength of this association compared with more obligately associated partners (e.g. cattle and their ruminal bacteria, insects with obligate endosymbionts). While our data cannot speak to the strength of association between bats and their bacteria, we suggest that selection on the microbiome may act more at the level of metabolic functions than on bacterial taxonomy. This interpretation may help to explain why bats have such high inter-individual variation in microbiome taxonomic composition. In addition, bat longevity may play a role in generating such variation. Bats are incredibly long lived for their body sizes [50], which may allow them to more thoroughly sample their environment for microbes throughout the course of their lifetimes. This hypothesis would help explain why bats show strong geographic patterning in microbiome taxonomic composition [51]. Few studies explicitly track individual bat microbiome turnover through time, and field studies on age-related phenomena in bats are limited due to the logistical challenges of recapturing individuals throughout their long adult life stages. Longitudinal studies of the microbiomes of laboratory-kept individuals could potentially test hypotheses about the stability of these relationships through time and with changing diets, adding valuable insight to the dynamics of bat-microbiome symbioses.

## Conclusions

Our study found differential enrichment in predicted bacterial metabolic pathways—including essential amino acid synthesis, fatty acid biosynthesis, and the generation of cofactors and vitamins essential for proper nutrition—across bat dietary guilds. These results represent novel insights into potential metabolic collaborations between gut microbes and their wild mammalian hosts. Future studies can add further depth and resolution to the patterns we identified here by including more direct functional inference methodologies, such as shotgun metagenomics and metatranscriptomics. In addition, experimental approaches using metabolomic tools can be used to further partition the nutrient landscape of mammals between endogenously synthesized products and those provisioned by symbiotic gut bacteria. Our results, which cover a large proportion of extant bat diversity, provide novel functional insights into an ecologically and evolutionarily rich host-microbe system.

## Methods

### Data collection

For this meta-analysis, we combined three bat microbiome data sets, two of which were previously published and one that was generated as part of this study (Additional file 3: Table S2). The 16S rRNA gene data for African bats were downloaded from the QIITA database from a study conducted by Lutz et al. 2019 [11]. This dataset contained 402 guano samples (31 species), and was prepared according to the Earth Microbiome Project protocols targeting the V4 region of the 16S rRNA gene (515f/806r) [52]. We also included previously published vampire bat microbiotas from Ingala et al. (2019) (*n* = 23, 1 species) to increase ecological coverage, which also included 16S genes sequenced using the V4 region (515f/806r) [25].

New data were generated from fecal samples of bat species from the Americas captured in and around the Lamanai Archaeological Reserve in Orange Walk District, Belize (17.75117° N, 88.65446° W) in April–May of 2016, 2017, and 2018 (*n* = *114, *28 species). During field sampling, we adhered to the best practices for humane capture and handling of live mammals outlined by the American Society of Mammalogists [53], and all field protocols were approved by institutional animal care and use committees at the American Museum of Natural History (AMNH) IACUC-20180123 and Southern Connecticut State University (SCSU) IACUC S15-01.18. Briefly, bats were live captured in ground-level mist nets or harp traps and placed into individual clean cloth holding bags. Fecal samples were collected directly from bats or from the bottom of holding bats within 30 min of defecation using sterilized forceps. Each sample was placed into a sterile barcoded tube and immediately preserved in liquid nitrogen. Between uses, holding bags were washed in an industrial laundry to minimize cross-contamination of fecal samples, and forceps were twice sterilized between uses with a 10% DNA-Away solution (Molecular Bioproducts, Inc., San Diego, CA) and water. Samples were shipped frozen to the AMNH and stored at − 80 °C prior to DNA extraction.

### Dietary classification scheme

Because of the limited within-guild sample sizes for some dietary categories, such as carnivores, bats were classified into both “coarse” (Animalivorous or Herbivorous) and “fine” (Frugivorous, Carnivorous, Insectivorous, Sanguivorous, Omnivorous) dietary categories for statistical testing. This classification scheme was based on a thorough review of recent literature, taking into account newer diet studies that have overturned or expanded previous assumptions about host diet [e.g., 25, 26]. Still, many species do not fit neatly into dietary guilds because their feeding habits vary seasonally during breeding or in response to resource availability [33, 40, 54–55]. We therefore collected species-level foraging information from the EltonTraits database [56]. This database splits the overall resource use for each species into various percentages of fruit and nectar, vertebrate prey, and insects, and may therefore be a more ecologically realistic method of measuring the feeding niches of the species in this study. We also used this database to validate fine-scale niche assignments, such that bats assigned to a fine-scale category had to have at least 50% of their diet comprised of a single type of resource, and any bats whose diets were composed of approximately equal plant and animal material were assigned as “omnivores”(e.g., *Phyllostomus discolor*, whose diet is coded as 30% invertebrates, 40% fruit, and 30% nectar in EltonTraits).

### DNA extraction

We performed all DNA isolations and library preparations in a UV-sterilized laminar flow hood to prevent aerosol contamination. We extracted total DNA from each guano sample using the QIAamp PowerFecal DNA Kit (MO BIO Laboratories, QIAGEN Co., Carlsbad, CA) following the manufacturer’s instructions with the following alterations: prior to homogenization, we incubated fecal samples in the provided lysis solutions (Powerbead + Solution C1) for 10 min at 70 °C. Next, we homogenized the fecal material in a Fisherbrand Bead Mill 24 homogenizer (Fisher Scientific, Pittsburgh, PA) at 6 m/s for 1–2 min, until the fecal slurry was fully homogenized. At the elution step, we eluted with warmed PCR-grade water instead of the provided C6 buffer and incubated columns for two minutes prior to centrifugation. In addition to our samples, we extracted one “blank” (water only) sample to account for bacterial contamination of the extraction kit, which has been documented as an important source of error in other metagenomic studies [57, 58]. As a positive control, we also extracted 25 µL of genomic DNA from a mock microbial community of known composition (ZYMOBIOMICS, Zymo Research, Inc., Irvine, CA). Purified DNA extracts were preserved at − 25 °C prior to next generation sequencing (NGS) library preparation.

### Microbiota profiling

For the Belize 2016–2017 samples, libraries targeting the V4 hypervariable region of the 16S rRNA gene were amplified using primer pair 515F/806 [59, 60]. Amplicon libraries were generated and sequenced by MrDNA, using a single-end sequencing on an Illumina MiSeq platform (Shallowater, TX, USA). All 2018 fecal microbiome libraries were prepared and sequenced by the Integrated Microbiome Resource facility of Dalhousie University (Halifax, NS, Canada). Briefly, each 2018 fecal sample underwent PCR amplification of the V6-V8 hypervariable region of the 16S rRNA gene using universal primers 969FB and 1406R [61]. Both 2016–2017 and 2018 libraries were paired-end sequenced (2 × 300 bp) on an Illumina MiSeq platform using V3 chemistry. While it is generally preferable to standardize primer target regions, our data were prepared for other studies by independent contributors and later collated for meta-analysis. Different primer regions have the ability to produce slightly different taxonomic assemblages, but in general, beta diversity metrics have been shown to be robust to both primer region and sequencing platform biases [62].

### Functional profiling

We processed data generated from different sequencing runs separately using the QIIME2 v. 2019.10 pipeline of tools [63] We imported each dataset and performed quality filtering with the DADA2 plugin, which trims barcode and primer sequences, identifies and filter chimeric sequences, and calls amplicon sequence variants (ASVs) [64]. In general, we trimmed the first 10–20 base pairs to account for low-quality reads and truncated each dataset at the point where per-base quality score tapered to below roughly Q = 20. We then used the representative sequences as input for taxonomic classification using the naïve Bayesian classifier trained on the SILVA 132 99% OTUs database [65, 66]. Each classifier was individually trained on the specific primer sets used in each study as recommended by the developers [67]. Because each dataset was prepared with a slightly different set of genetic protocols, we processed each one separately until taxonomic assignment was determined. After generating taxonomic feature tables for each dataset, we further filtered out mitochondrial and chloroplast reads from the datasets as well as any reads that could not be defined at least to the phylum level. Such sequences may represent novel bacteria not yet characterized in 16S rRNA gene databases and are of limited use for functional prediction, since functionally annotated whole genome databases are generally less complete than marker gene databases [68]. After filtering, all datasets were merged into a single feature table for functional profiling.

It is not possible to directly infer bacterial functions from marker gene inventories, so we used Phylogenetic Investigation of Communities by Reconstruction of Unobserved States (PICRUSt2) to predict metagenomic profiles for each microbiota sample [69, 70]. PICRUSt2 works by first inserting observed 16S rRNA gene sequences into a bacterial reference phylogeny, and then using hidden state prediction models to assign functions based on the closest matching bacterial reference genome [70]. The output of the algorithm reports an ASV abundance table normalized by predicted 16S rRNA gene copy number for each ASV. We merged all ASV tables prior to PICRUSt2 inference to ensure that the same predictions would be output for the same ASVs present across multiple feature tables.

### Statistical analyses

Previous studies suggest that rarefying data to account for variable library depth is not appropriate [71], so instead of rarefying our data to an arbitrary subsampling depth, we performed a Hellinger transformation to scale the data using R package *microbiome* [72, 73]. We first tested if overall metagenome functional profiles differed according to host taxonomy and dietary ecology (both coarse and fine) using the adonis.pair function in R package *EcolUtils* and applying a Benjamini–Hochberg correction for multiple comparisons [74]. Using R package *phyloseq* [75], we performed PERMANOVA tests on Bray–Curtis distances of metagenomes as a function of host identity and diet, taking into account the nested nature of host taxonomy [study.bray ~ FeedingNiche * HostSpecies + HostGenus + HostFamily]. Next, we performed paired PERMANOVAs to test for differences between each individual feeding niche.

PERMANOVA can detect differences between groups of data, but the test operates on distance matrices and therefore cannot determine which specific functions are driving group differences. To test for differential enrichment of specific metagenome functions, we performed Linear Discriminant Analysis Effect Size (LEfSe) analysis as implemented on the Galaxy platform (https://huttenhower.sph.harvard.edu/galaxy/) [76]. We grouped samples by feeding niche in both coarse (animalivores, herbivores) and fine (frugivores, insectivores, omnivores, carnivores, and sanguivores) ecological classification schemes, and set the LDA score cutoff to 2.5 to impose a strict effect size criterion on differentially abundant features. Due to the low number of omnivore observations, we grouped them together with the animalivorous bats for the coarse LEfSe analysis based on prior knowledge that these species rely heavily on insects during some seasons [39, 42, 77].

We also sought to assess the influence of diet and host phylogeny on predicted microbiome functions by representing these values as continuous traits. We merged metagenome functions by host species and computed the Bray–Curtis distances for all species. For the host phylogeny, we computed patristic distances (i.e., the sum of the branch lengths linking two nodes) between terminal taxa using a pruned phylogeny from Upham et al. 2019 [78]. We reconciled taxonomic changes between the sampled species and their closest synonymous or sister taxon represented in the Upham dataset using batnames.org (Additional file 2: Table S1) [79]. For each of these same taxa, we also collected species-level dietary data from the EltonTraits database [56], which represents mammalian diets as percentages of various food resources (vertebrates, insects, nectar, fruit, etc.). We transformed these proportional data into a distance matrix using the function “dist.prop” in R package *ade4* using the “Manly” method [80, 81]. Using these matrices, we tested for associations between gut microbiome functions and host phylogeny and diet using multiple regression on matrices (MRM) implemented in the R package *ecodist* using the formula merged.functional.dist ~ bat.diets.dist + PatristicDistMatrix [82]. Because bat microbiomes are known to be highly variable among individuals of the same species [11, 43], we also tested the predictive power of host diet using random forests on the full per-individual dataset. We first removed any features from the dataset that were present in fewer than 10% of samples and scaled all raw counts by transforming to Z-scores. Finally, we constructed random forest classifiers using R package *randomForest* to test the ability of the functional profiles of each sample to predict the coarse or fine niche of the host [83, 84]. Each classifier was built over 10,000 trees and out-of-bag error rate (OOB%) was estimated for each model. Model significance and accuracy was further evaluated using permutation testing and cross-validation, respectively.

We tested for evolutionary signal in microbiome functions by treating each discriminatory functional pathway identified by the random forest analysis as a trait of the host following an approach similar to that used by Capunitan et al. (2020) [47]. We used a pruned species-level phylogeny of bats from Upham et al. 2019 [78]. Because host taxonomy changes frequently, we manually correct species names to reflect the most up-to-date taxonomy and to reconcile ambiguous host species identities (Additional file 2: Table S1). Microbiome traits were averaged across individuals of the same species using “merge_samples” prior to undergoing center-log transform and matched to the tips in the phylogeny using the “treedata” function. Using the “fitContinuous” function in *geiger* [85], we tested the fit of Brownian Motion, Ornstein–Uhlenbeck (OU; single optimum), Early Burst, and White Noise models and compared them using weighted Akaike information criterion (AIC). Akaike weights were calculated from AIC scores using the “aicw” function. As a measure of phylogenetic signal, we calculated Pagel’s lambda (λ) [86], which is a scaling parameter that ranges from 0 (no phylogenetic signal) to 1 (strong phylogenetic signal).

## Supplementary Information

**Additional file 1: Fig. S1.** Principal coordinates analysis of predicted bat microbiome functions colored according to coarse host niche.

**Additional file 2: Table S1.** Table of species sampled in this study and their closest relative in the Upham et al. 2019 phylogeny. Patristic distances were computed using the closest terminal taxon available in this phylogeny, and taxon names for the MRMs were coerced to match those in the phylogeny. Two species in the Lutz et al. dataset were not identified beyond genus; for these, we chose a congeneric species known to occur in the sampled localities for use in patristic distance calculations.

**Additional file 3: Table S2.** Sample metadata and study provenance for all bat microbiomes used in this study.

**Additional file 4: Table S3.** Confusion matrix for Random Forest models attempting to predict host family membership based on metagenome functions. Out-of-bag error rate was 49.36%.

**Additional file 5: Table S4.** Confusion matrix for Random Forest models attempting to predict host genus membership based on metagenome functions. Out-of-bag error rate was 58.35%.

## Data Availability

All Belize 2016, 2017, and 2018 raw 16S rRNA gene sequences are publicly available at the NCBI SRA under BioProject PRJNA701438. Statistical packages and full code are available at https://github.com/MelissaIngala/BatFunx. Additional metadata files can be accessed on FigShare:(https://figshare.com/projects/You_Are_More_Than_What_You_Eat_Differential_Enrichment_of_Microbiome_Functions_Across_Bat_Dietary_Niches/98408). Data from previous studies has been made publicly available by the authors.

## Abbreviations

AMNH: American Museum of Natural History
ASV: Amplicon sequence variant
BM: Brownian Motion
EB: Early Burst
LefSE: Linear discriminant analysis Effect Size
MRM: Multiple regression on matrices
NGS: Next-generation sequencing
OOB: Out-of-bag error
OU: Ornstein-Uhlenbeck
PERMANOVA: Permutational multivariate analysis of variance
