# Supplementary figures and images for: You are more than what you eat: potentially adaptive enrichment of microbiome functions across bat dietary niches
Source: Anim Microbiome. 2021 Dec 14;3:82. doi: 10.1186/s42523-021-00139-8 (PMC8672517; doi:10.1186/s42523-021-00139-8)

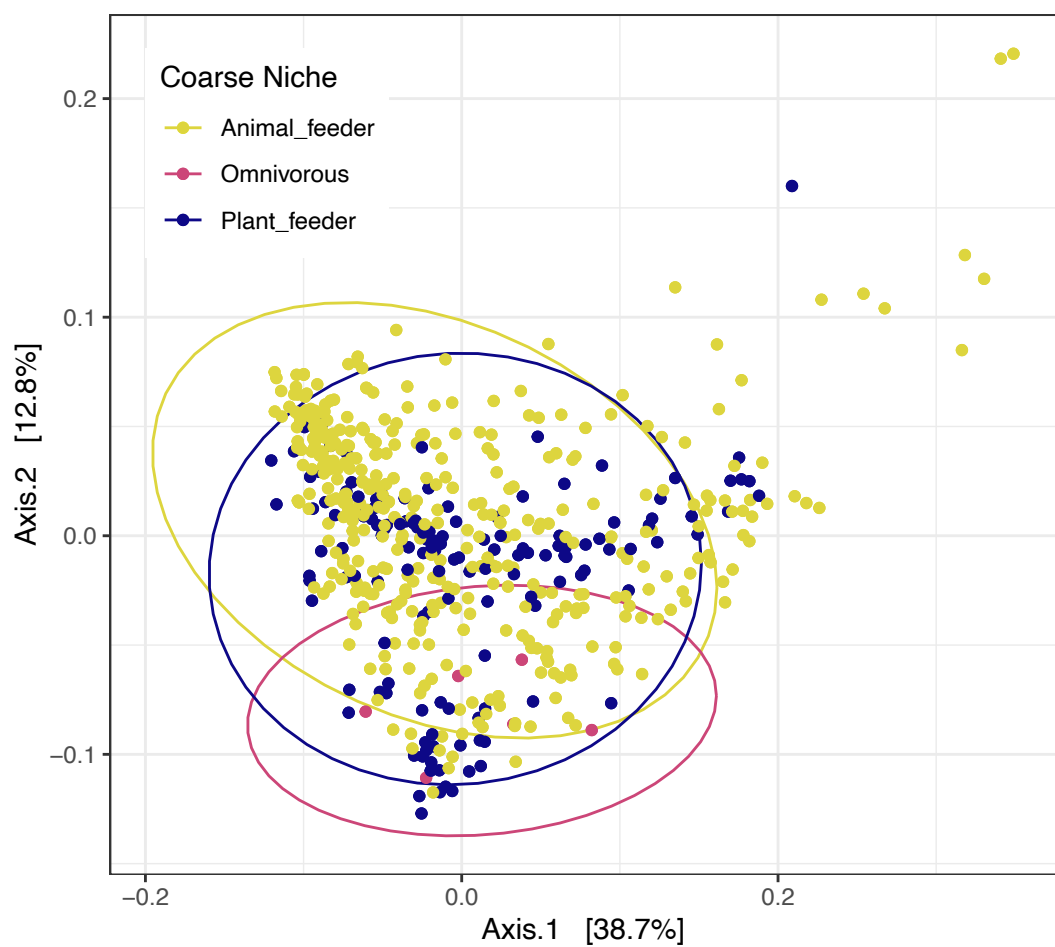

Supplement: Supplementary file 1 — Additional file 1: Fig. S1. Principal coordinates analysis of predicted bat microbiome functions colored according to coarse host niche. [file 42523_2021_139_MOESM1_ESM.pdf]
