# Supplementary material for: You are more than what you eat: potentially adaptive enrichment of microbiome functions across bat dietary niches
Source: Anim Microbiome. 2021 Dec 14;3:82. doi: 10.1186/s42523-021-00139-8 (PMC8672517; doi:10.1186/s42523-021-00139-8)
Supplement: Supplementary file 2 — Additional file 2: Table S1. Table of species sampled in this study and their closest relative in the Upham et al. 2019 phylogeny. Patristic distances were computed using the closest terminal taxon available in this phylogeny, and taxon names for the MRMs were coerced to match those in the phylogeny. Two species in the Lutz et al. dataset were not identified beyond genus; for these, we chose a congeneric species known to occur in the sampled localities for use in patristic distance calculations. [file 42523_2021_139_MOESM2_ESM.docx]

**Supplementary Table S1**

| **Taxon names as they appear in original microbiome studies** | **Representative taxon in Upham et al. 2019 phylogeny** |
| --- | --- |
| Gardnernycteris_crenulatum | *Mimon_crenulatum* |
| Mormoops_megalophylla | *Mormoops_megalophylla* |
| Bauerus_dubiaquercus | *Bauerus_dubiaquercus* |
| Eptesicus_furinalis | *Eptesicus_furinalis* |
| Artibeus_lituratus | *Artibeus_lituratus* |
| Artibeus_jamaicensis | *Artibeus_jamaicensis* |
| Glossophaga_soricina | *Glossophaga_soricina* |
| Carollia_sowelli | *Carollia_sowelli* |
| Carollia_perspicillata | *Carollia_perspicillata* |
| Rhogeessa_aeneus | *Rhogeessa_aeneus* |
| Molossus_nigricans | *Molossus_rufus* |
| Pteronotus_personatus | *Pteronotus_personatus* |
| Mimon_cozumelae | *Mimon_cozumelae* |
| Chrotopterus_auritus | *Chrotopterus_auritus* |
| Trachops_cirrhosus | *Trachops_cirrhosus* |
| Dermanura_watsoni | *Dermanura_watsoni* |
| Saccopteryx_bilineata | *Saccopteryx_bilineata* |
| Myotis_pilosatibialis | *Myotis_keaysi* |
| Lasiurus_ega | *Lasiurus_ega* |
| Myotis_elegans | *Myotis_elegans* |
| Pteronotus_mesoamericanus | *Pteronotus_parnelli* |
| Dermanura_phaeotis | *Dermanura_phaeotis* |
| Artibeus_intermedius | *Artibeus_planirostris* |
| Lophostoma_evotis | *Lophostoma_evotis* |
| Sturnira_parvidens | *Sturnira_parvidens* |
| Natalus_mexicanus | *Natalus_mexicanus* |
| Rhynchonycteris_naso | *Rhynchonycteris_naso* |
| Noctilio_leporinus | *Noctilio_leporinus* |
| Desmodus_rotundus | *Desmodus_rotundus* |
| Phyllostomus_discolor | *Phyllostomus_discolor* |
| Pteronotus_fulvus | *Pteronotus_davyi* |
| Chaerephon_bivitattus | *Chaerephon_bivitattus* |
| Epomophorus_labiatus | *Epomophorus_labiatus* |
| Micropteropus_pusillus | *Micropteropus_pusillus* |
| Nycteris_arge | *Nycteris_arge* |
| Miniopterus_natalensis | *Miniopterus_natalensis* |
| Rhinolophus_clivosus acrotis | *Rhinolophus_clivosus* |
| Rousettus_aegyptiacus | *Rousettus_aegyptiacus* |
| Myotis_tricolor | *Myotis_tricolor* |
| Epomophorus_wahlbergi | *Epomophorus_wahlbergi* |
| Stenonycteris_lanosus | *Rousettus_lanosus* |
| Myonycteris_angolensis | *Myonycteris_angolensis* |
| Scotoecus_hindei | *Scotoecus_hirundo* |
| Pipistrellus_sp. | *Pipistrellus_nanulus* |
| Otomops_harrisoni | *Otomops_ martiensseni* |
| Rhinolophus_eloquens | *Rhinolophus_eloquens* |
| Rhinolophus_clivosus | *Rhinolophus_clivosus* |
| Triaenops_afer | *Triaenops_afer* |
| Miniopterus_minor | *Miniopterus_minor* |
| Miniopterus_africanus | *Miniopterus_fuliginosus* |
| Hipposideros_caffer | *Hipposideros_caffer* |
| Coleura_afra | *Coleura_afra* |
| Macronycteris_vittatus | *Hipposideros_vittatus* |
| Neoromicia_sp. | *Neoromicia_capensis* |
| Scotophilus_dinganii | *Scotophilus_dinganii* |
| Rhinolophus_landeri | *Rhinolophus_landeri* |
| Nycteris_thebaica | *Nycteris_thebaica* |
| Hipposideros_ruber | *Hipposideros_ruber* |
| Miniopterus_inflatus rufus | *Miniopterus_inflatus* |
| Doryrhina_camerunensis | *Hipposideros_camerunensis* |
| Neoromicia_nana | *Neoromicia_nana* |
