# Supplementary material for: You are more than what you eat: potentially adaptive enrichment of microbiome functions across bat dietary niches
Source: Anim Microbiome. 2021 Dec 14;3:82. doi: 10.1186/s42523-021-00139-8 (PMC8672517; doi:10.1186/s42523-021-00139-8)
Supplement: Supplementary file 3 — Additional file 3: Table S2. Sample metadata and study provenance for all bat microbiomes used in this study. [file 42523_2021_139_MOESM3_ESM.docx]

**Supplemental Table S3**

|  | Emballonuridae | Hipposideridae | Miniopteridae | Molossidae | Mormoopidae | Natalidae | Noctilionidae | Nycteridae | Phyllostomidae | Pteropodidae | Rhinolophidae | Rhinonycteridae | Vespertilionidae | class.error |
| --- | --- | --- | --- | --- | --- | --- | --- | --- | --- | --- | --- | --- | --- | --- |
| Emballonuridae | 4 | 4 | 4 | 0 | 0 | 0 | 0 | 0 | 5 | 1 | 0 | 1 | 0 | 0.79 |
| Hipposideridae | 0 | 27 | 14 | 8 | 0 | 0 | 0 | 0 | 6 | 8 | 9 | 1 | 1 | 0.64 |
| Miniopteridae | 0 | 17 | 44 | 4 | 0 | 0 | 0 | 0 | 9 | 4 | 11 | 1 | 1 | 0.52 |
| Molossidae | 0 | 9 | 9 | 14 | 0 | 0 | 0 | 0 | 7 | 2 | 4 | 0 | 1 | 0.70 |
| Mormoopidae | 0 | 0 | 1 | 0 | 5 | 0 | 0 | 0 | 4 | 2 | 0 | 0 | 1 | 0.62 |
| Natalidae | 0 | 0 | 1 | 0 | 0 | 0 | 0 | 0 | 1 | 0 | 0 | 0 | 0 | 1 |
| Noctilionidae | 0 | 0 | 0 | 1 | 0 | 0 | 0 | 0 | 0 | 0 | 0 | 0 | 0 | 1 |
| Nycteridae | 0 | 7 | 4 | 1 | 0 | 0 | 0 | 2 | 0 | 0 | 2 | 0 | 0 | 0.88 |
| Phyllostomidae | 1 | 1 | 4 | 1 | 0 | 0 | 0 | 0 | 87 | 4 | 0 | 0 | 1 | 0.12 |
| Pteropodidae | 0 | 3 | 2 | 1 | 0 | 0 | 0 | 0 | 6 | 75 | 4 | 0 | 0 | 0.18 |
| Rhinolophidae | 0 | 11 | 12 | 4 | 0 | 0 | 0 | 1 | 11 | 2 | 17 | 0 | 0 | 0.71 |
| Rhinonycteridae | 0 | 1 | 6 | 0 | 0 | 0 | 0 | 0 | 1 | 0 | 0 | 1 | 0 | 0.89 |
| Vespertilionidae | 0 | 2 | 5 | 1 | 1 | 0 | 0 | 0 | 12 | 3 | 2 | 0 | 0 | 1 |
